# Interactions and Insertion of *Escherichia coli* Hfq into Outer Membrane Vesicles as Revealed by Infrared and Orientated Circular Dichroism Spectroscopies

**DOI:** 10.3390/ijms241411424

**Published:** 2023-07-13

**Authors:** Florian Turbant, Jehan Waeytens, Anaïs Blache, Emeline Esnouf, Vincent Raussens, Grzegorz Węgrzyn, Wafa Achouak, Frank Wien, Véronique Arluison

**Affiliations:** 1 Laboratoire Léon Brillouin LLB, CEA, CNRS UMR12, Université Paris Saclay, CEA Saclay, 91191 Gif-sur-Yvette, France; florian.turbant@synchrotron-soleil.fr (F.T.);; 2 Synchrotron SOLEIL, L’Orme des Merisiers, Saint Aubin BP48, 91192 Gif-sur-Yvette, France; frank.wien@synchrotron-soleil.fr; 3 Department of Molecular Biology, University of Gdansk, Wita Stwosza 59, 80-308 Gdansk, Poland; grzegorz.wegrzyn@ug.edu.pl; 4 Structure et Fonction des Membranes Biologiques, Université libre de Bruxelles, 1050 Bruxelles, Belgium; jehan.waeytens@ulb.be (J.W.); vincent.raussens@ulb.be (V.R.); 5 Lab of Microbial Ecology of the Rhizosphere, (LEMiRE), BIAM, CEA, CNRS, Aix Marseille University, 13115 Saint Paul Lez Durance, France; anais.blache@cea.fr (A.B.); wafa.achouak@cea.fr (W.A.); 6 UFR Sciences du Vivant, Université Paris Cité, 75006 Paris, France

**Keywords:** bacterial communication, cross-kingdom RNA interference, outer membrane vesicle (OMV), Gram-negative bacteria, small noncoding regulatory RNA (sRNA), Hfq, bacterial amyloid, membrane insertion, lipid–protein interaction

## Abstract

The possible carrier role of Outer Membrane Vesicles (OMVs) for small regulatory noncoding RNAs (sRNAs) has recently been demonstrated. Nevertheless, to perform their function, these sRNAs usually need a protein cofactor called Hfq. In this work we show, by using a combination of infrared and circular dichroism spectroscopies, that Hfq, after interacting with the inner membrane, can be translocated into the periplasm, and then be exported in OMVs, with the possibility to be bound to sRNAs. Moreover, we provide evidence that Hfq interacts with and is inserted into OMV membranes, suggesting a role for this protein in the release of sRNA outside the vesicle. These findings provide clues to the mechanism of host–bacteria interactions which may not be defined solely by protein–protein and protein–outer membrane contacts, but also by the exchange of RNAs, and in particular sRNAs.

## 1. Introduction

Outer membrane vesicles (OMVs) are small spherical structures (~100 nm) released by Gram-negative bacteria. Observed for the first time in the 1960s [1], these vesicles play a significant role in bacterial communication, virulence, and in adaptations to changes in the environment [2]. Originating from the outer membrane (OM), they consist of proteo-liposomes; hence, their composition reflects the components of the outer membrane (i.e., mainly phospholipids, lipopolysaccharides (LPS), and outer membrane proteins). Internally, they also contain periplasmic and cytoplasmic components, referred to as cargoes. These include mainly proteins and nucleic acids.

As vehicles, OMVs serve the purpose of delivering various biomolecules to both host cells and other bacteria [3,4]. Functionally, OMVs are recognized as playing crucial roles in cell-to-cell communication. In particular, virulence factors and toxins are often found in OMVs. For instance, Shiga toxins, produced by enterohemorrhagic *Escherichia coli* (EHEC) strains, have been identified in the OMVs content [5].

Apart from proteins, small regulatory non-coding RNAs (sRNAs) have also been found in OMVs and classified as new OMV-associated virulence factors [4,6]. RNAs can indeed be found in different extracellular vesicles, in outer membrane vesicles (OMVs) but also in membrane vesicles (MV) for Gram-negative and Gram-positive bacteria, respectively [7].

sRNAs are usually ~100 nucleotides in length and are usually produced under specific stress conditions. They have multifaceted roles including post-transcriptional regulation, allowing stress responses and environmental adaptation [8,9]. sRNAs such as MicF can, for instance, bind to mRNAs, coding for proteins of the outer membrane, such as OmpF [10]. OMVs not only protect sRNAs from fast degradation outside of cells by RNAses, but also facilitate their transport to target cells, thus enabling intercellular communication and the modulation of gene expression in recipient cells [6,11]. Bacterial sRNAs have, for instance, been shown to modulate the expression of genes required for plant–bacteria interactions [12]. sRNAs are in addition implicated in the control of the content, biogenesis, and function of OMVs. For example, the overexpression of genes coding for sRNAs—such as MicA in *Salmonella enterica* and *E. coli*, and VrrA in *Vibrio cholerae*—regulate *ompA* and enhance OMV production [13,14]. These findings thus underscore the regulatory role of sRNAs in modulating OMV formation, in addition to their crucial roles as carriers of sRNAs, allowing a cross-talk between bacterial and eukaryotic cells, a process now referred as “cross-kingdom RNA interference” [15,16].

Interestingly, sRNAs usually require a protein cofactor, named Hfq, for their regulatory functions [8,17]. Hfq serves as a crucial chaperone in sRNA-mediated regulation in bacteria. By binding to sRNAs, Hfq stabilizes them and facilitates their interactions with target mRNAs. This interaction leads to post-transcriptional regulation through processes such as mRNA degradation or translational control. Structurally, Hfq forms a torus-shaped hexamer, with six C-terminal regions (CTRs) extending outward from the core structure [17]. Nevertheless, the role and structure of this elongated C-terminal region are still not fully understood, with the notable exception of its ability to form an amyloid-like assembly [18]. Furthermore, while the presence of sRNAs inside OMVs has been established [6,16], the presence of Hfq remains uncertain. However, given its ability to interact with and permeate bacterial inner membranes in vitro [19,20], it is plausible that Hfq may be present in the periplasmic space and subsequently exported in OMVs with sRNAs.

In this study, we have expanded on our previous findings by demonstrating the association of Hfq not only with the inner membrane (IM) [19,20], but also with the outer membrane (OM). This interaction between Hfq and the outer membrane vesicle is significant as it may play a crucial role in sRNA-based regulation [21,22,23]. The purpose of the present study was thus to use a combination of ATR-FTIR and synchrotron radiation circular dichroism (SRCD) spectroscopies, which are two fast and complementary methods for amyloid–OMV interaction analysis. This analysis is challenging due to the complexity of the OMV membrane components, particularly the presence of lipopolysaccharides (LPS), which poses a challenge for methods observing protein insertion into membranes, such as polarized FTIR or AFM [24,25]. To address this important biological question, we employed a combination of vibrational and electronic absorption spectroscopies to provide insights into Hfq–OM interactions. Vibrational spectroscopy and more specifically infrared spectroscopy can be used to study the conformational changes of proteins in the presence of lipids and OMV, while Orientated SRCD (OCD) gives information about the insertion of the protein in OMV [20]. In the present work, we focused our attention on conformational changes observed in Hfq during its incubation in the presence of OMVs to shed light on the insertion of the protein in the membrane.

## 2. Results

### 2.1. Hfq Is Present in OMV

**Figure 1:**
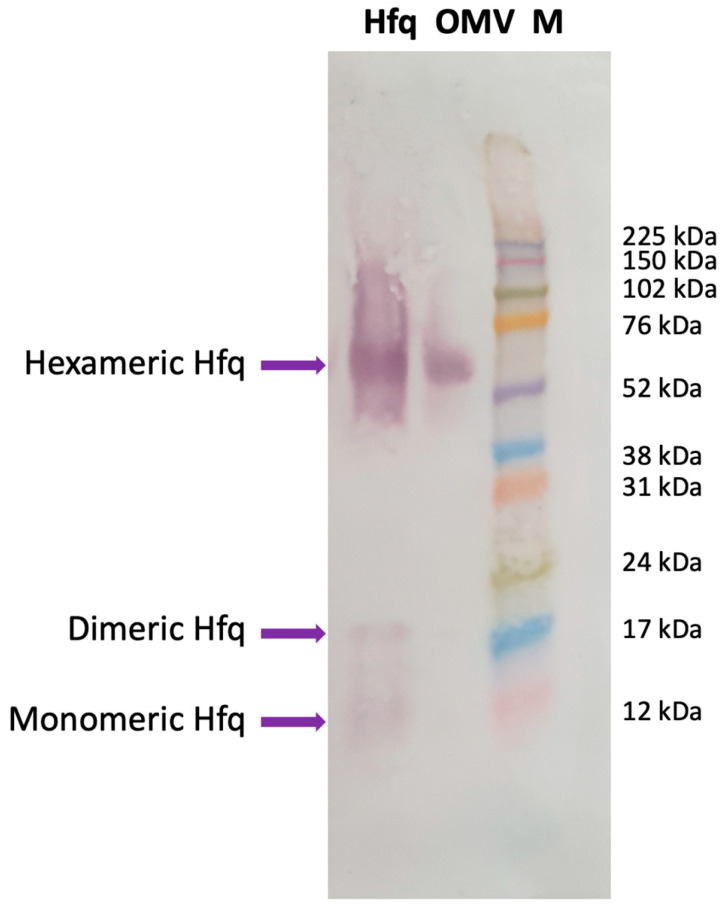
Western blotting analysis showing the presence of Hfq in OMVs extracted from wild-type *E. coli*. Hfq: purified Hfq; OMV: OMV purified from *E. coli* WT strain; M: molecular-weight marker (Amersham ECL Rainbow full range). The membrane was successively incubated with a goat anti-Hfq polyclonal antibody (dilution 1/1000, Origene, Herford, Germany), with an anti-goat secondary antibody coupled to alkaline phosphatase (dilution 1/1000, Sigma, Saint Louis, MO, USA); the phosphatase was revealed using a BCIP/NPT solution. Purified Hfq was analyzed as a positive control for the position of migration. The Anti-Hfq antibodies recognize mainly the hexameric form of the protein in OMVs, as previously described [19,26,27]. Note that we revealed the membrane for a long time to observe, on the same blot, the monomeric and dimeric forms of Hfq, clearly observed with purified Hfq and to a lower extent in OMVs [19].

Our previous analyses showed that full-length Hfq and Hfq-CTR both interact with a lipid bilayer mimicking the *E. coli* inner membrane, with the concomitant formation of holes in the membranes [19,20]. This raised the possibility that Hfq (and associated sRNA) may be translocated into the periplasm, and may possibly remain in contact with the OM. While the presence of sRNAs inside OMVs has been clearly established previously [6,16], the presence of Hfq still remains uncertain. For this reason, we first analyzed OMV content via Western blotting using anti-Hfq antibodies. As shown in Figure 1, the presence of Hfq can be clearly demonstrated in the OMV, indicating that the protein is transiently present in the periplasm and is then exported into the OMV as a cargo.

### 2.2. Hfq Physically Interacts with OMV

OMVs are composed of different lipids, and, in particular, LPS of various lengths. An analysis using molecular imaging, such as atomic force microscopy to observe the insertion of a protein into the OM membrane, is thus usually difficult [25]. We thus proposed two alternative spectroscopic methods, Fourier-transform infrared (FTIR) spectroscopy and orientated synchrotron radiation circular dichroism (OCD), to follow the interaction between Hfq and OMVs (extracted from a ∆*hfq* strain) and to observe the possible insertion of Hfq into OMVs. The combination of these two techniques allowed us to obtain evidence for the effect of OMV membranes on the protein structure and Hfq insertion into the OMV membrane [24].

**Figure 2:**
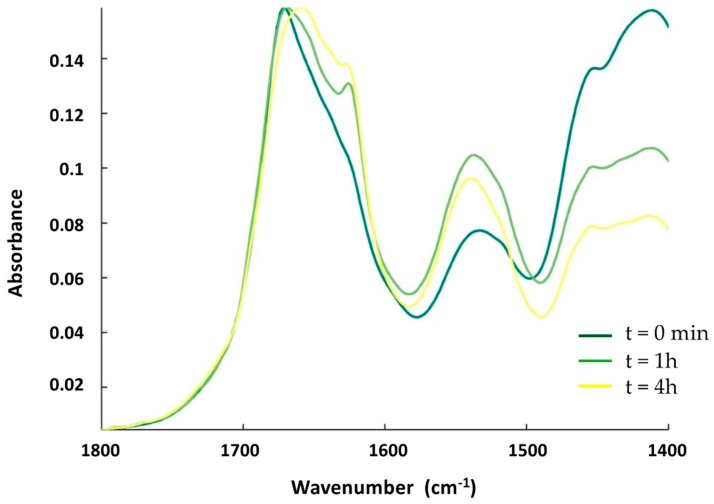
ATR-FTIR kinetics of the interaction between Hfq and OMV. The spectra (from dark-green to yellow) are taken at t = 0 min, t = 1 h, and t = 4 h. The band at 1610 cm^−1^, characteristic of amyloid fibrils, increases during the interaction of the protein with OMV during the first part of the incubation (one hour, compare dark-green and light-green spectra) and then decreases (compare light-green and yellow spectra).

First, we tested the effect of OMVs on the structure of Hfq using FTIR spectroscopy. OMVs were purified from the ∆*hfq* strain to avoid the presence of endogenous Hfq prior to analysis. OMVs extracted from the ∆*hfq* strain were first observed using transmission electron microscopy (TEM) and negative staining [20] to confirm their homogeneity and the absence of contaminants (Supplementary Figure S1). To allow them to interact, the OMVs and Hfq (in deuterated buffer) were mixed together and incubated in solution. The kinetics of the reaction were measured by taking a few µL of the OMV–Hfq mixture at different times; this was then deposited on an ATR (attenuated total reflection) surface, in this case a diamond crystal. The sample was dried with nitrogen as water (present in OMV) absorbs strongly in the same region as the amide I band of the proteins (~1650 cm^−1^). After evaporation, ATR-FTIR spectra were recorded. It should be noted that with ATR-FTIR, the penetration depth is around 1 µm; therefore, only molecules close to the surface are observed. Figure 2 shows the amide I’ band of Hfq (amide I’ refers to amide I bands in D_2_O, as the Hfq protein was in deuterated buffer). The β-sheets involved in the amyloid structure usually absorb between 1630 and 1610 cm^−1^ in the IR region [28]. For Hfq, the position of the β-sheet amyloid is around 1610 cm^−1^ (Figure 2), in agreement with previous reports [29,30,31]. The evolution of the Hfq secondary structure can be followed by looking at the amide I’ band signal [32]. Figure 2 shows the kinetics of the interaction between OMV and Hfq. Precisely, at the beginning of the kinetic analysis, we observed two major bands in the amide I’ region: at 1660 cm^−1^, corresponding to random and α-helix structures, and a second one at 1610 cm^−1^, corresponding to the amyloid β-sheet. We observed an important increase in the 1610 cm^−1^ peak during the first hour of incubation, indicating a rapid conformational change with an increase in the amyloid-like β-sheet structure. Furthermore, we observed, via FTIR, a rising band at ~1650 cm^−1^, indicating an increase in the random structure, and, after 4 h of kinetics, this became the most intense signal. This result thus indicates that OMV enhances the amyloidogenicity of Hfq when they interact with each other. Note that the effect of lipids on Hfq amyloidogenicity has already been reported previously [20]. In addition, possibly when the protein is inserted in the OMV (see below), the amyloid structure is lost and converted into disordered structures.

**Figure 3:**
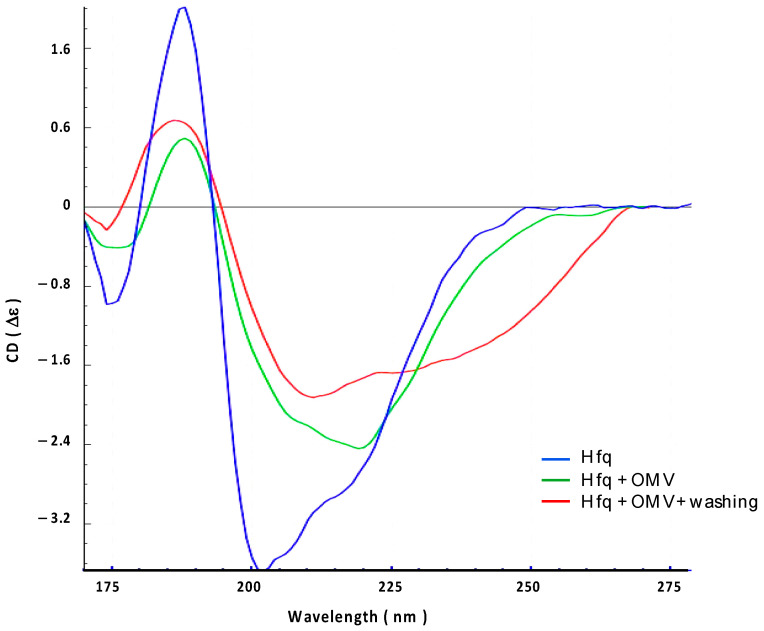
Orientated synchrotron radiation circular dichroism (OCD) analysis of the interaction between full-length Hfq and OMV-supported membrane. Blue: Hfq alone; Green: Hfq in contact with OMV for 200 min; Red: Hfq in contact with OMV after washing to remove excess on Hfq.

We then confirmed this result using synchrotron radiation circular dichroism (SRCD) (Figure 3). The secondary structure content of the Hfq protein prior to its interaction with the OMV membrane was analyzed using BeStSel [33], indicating that Hfq contains ~10% of the α-helix, ~25% of the β-sheet, and ~65% of other structures (turn or random) (Figure 3, blue spectrum). This composition is in agreement with previous crystallographic structures of *E. coli* Hfq, taking into account that all Hfq structures in PDB lack the CTR part of the protein when they have one (see PDB code 1HK9, 4V2S, 5UK7, or 3QHS as examples of *E. coli* Hfq structures). To analyze the effect of OMV on the Hfq structure, purified OMVs were deposited on a CaF_2_ surface. The transparency of the surface to UV, especially in the far-UV region, was first confirmed. The protein was then added to the surface. Taking into account the FTIR results (Figure 2), we suspect that OMV lipids may induce the self-assembly of Hfq and possibly its alignment; thus, we used orientated circular dichroism (OCD) [34]. The OCD technique obviates the effects of alignment (as in the case of an amyloid) in the CD spectra, which distorts the measurement due to linear dichroism absorption. The OCD spectra are obtained by averaging the SRCD spectra obtained at the different rotation angles of the beam-centered cell. As shown in Figure 3, we observed that after 200 min of contact with the membrane (green curve), in agreement with the conclusion of the FTIR results (Figure 2), the secondary structure of Hfq changed and a shoulder at ~220 nm, characteristic of amyloid structures, appeared [35,36]. Both interacting and non-interacting Hfq were analyzed under these conditions. We then rinsed the OMV surface to observe only interacting Hfq (Figure 3, red curve). The shoulder at 225 nm confirms the presence of a residual amyloid structure. A secondary structure analysis (BeStSel) indicated that the β-sheet content decreased by 15%, while that of other structures, such as the turn and random coil, increased. This result is in agreement with the FTIR results.

### 2.3. Hfq Inserts Inside OMV Membrane

**Figure 4:**
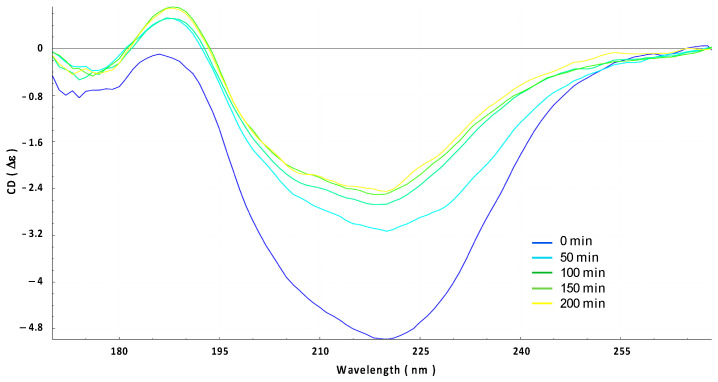
Orientated circular dichroism (OCD) evolution of the interaction between Hfq protein and OMV membrane for 200 min (from blue t = 0 min to yellow t = 200 min). Note that the yellow curve here corresponds to the green curve in Figure 2.

The insertion of Hfq into the OMV membrane was also analyzed using OCD kinetics. If the orientation of the protein changes from lying on the membrane (parallel) to being inserted into the membrane (perpendicular), the OCD signal should decrease significantly [24,34]. In other words, a reduction in the circularly polarized light absorption due to changes in the transition dipoles in the peptide bonds (n-π* and π-π* transitions) is responsible for the variation in the OCD signal amplitude. In Figure 4, we clearly observe that OCD amplitudes progressively decrease when Hfq interacts with the OMV membrane. This indicates that the protein is thus inserted into OMV.

### 2.4. Hfq-CTR Amyloid-like Structure Interacts with OMV and OMV Induces the Disassembly of the CTR

**Figure 5:**
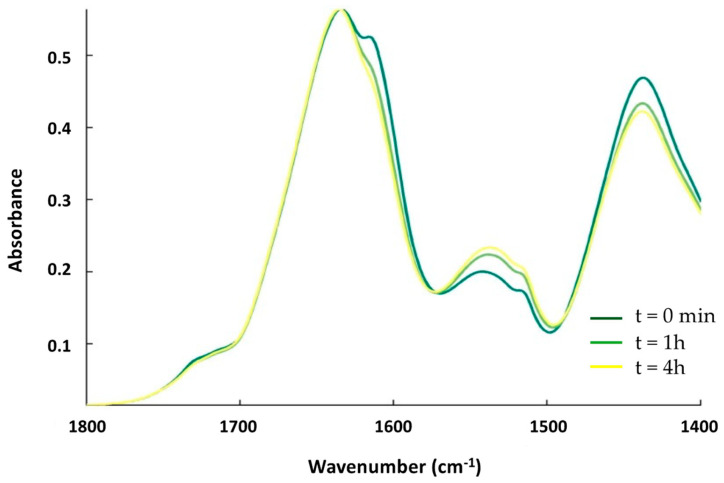
ATR-FTIR kinetics of the interaction between Hfq-CTR and supported OMV. The spectra from dark-green to yellow were also taken at t = 0 min, 1 h, and 4 h. Similar to full-length Hfq, the band at 1610 cm^−1^ first increases during the contact of the protein with OMV and then decreases.

The contact with OMV promotes the formation of an amyloid-like structure in the full-length Hfq followed by the disassembly of this structure when the protein is inserted in the OMV membrane. We wanted to confirm that this effect is due to the Hfq C-terminus region (CTR). We therefore analyzed the OMV interaction with pre-polymerized Hfq-CTR (devoid of the Sm toroidal core [37]). Figure 5 shows the kinetics of the interaction over 4 h. At t = 0 (dark green), the peak corresponding to the amyloid assembly is clearly detected at 1610 cm^−1^. Then, after one hour of interaction, the OMVs induce a loss of this peak and a disassembly of the amyloid structure, a similar behavior to that of full Hfq, as shown in Figure 2. This effect remains for at least 4 h.

**Figure 6:**
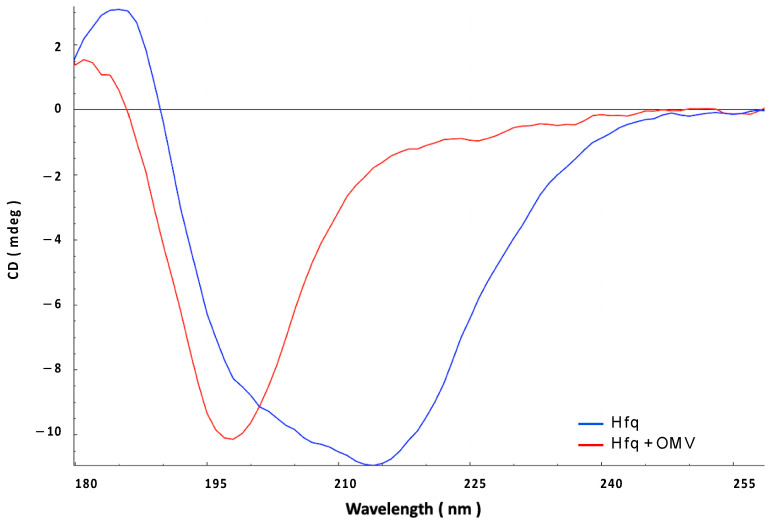
Synchrotron radiation circular dichroism (SRCD) analysis of the interaction between Hfq-CTR and OMV. Blue: pre-polymerized Hfq alone; red: Hfq in contact with OMV for 90 min.

This result was again confirmed via SRCD analysis (Figure 6). We observed that Hfq-CTR before the interaction presented a shoulder at ~220 nm, characteristic of an amyloid-like structure (blue spectrum) [36]. After 90 min of contact with the OMV, this shoulder at ~220 nm disappeared to give a peak at ~200 nm, corresponding to a disordered structure. This result is in agreement with FTIR results, confirming that the OMV membranes first promote amyloid formation, followed by the disassembly of CTR amyloid, similar to what we observed for full-length Hfq.

## 3. Discussion

The Hfq protein of *E. coli* is a relatively small polypeptide composed of 102 amino acid residues (aa), which interacts with different transcripts, in particular small noncoding RNAs, and functions as an RNA chaperone [38,39]. This activity operates predominantly through facilitating RNA–RNA interactions, thus modulating the availability of mRNA molecules to the translation machinery [9]. As such, it is involved in the control of the expression of various genes. Hfq is composed of two domains, called the N-terminal (NTR) and the C-terminal regions (CTR). NTR (~70 aa) is believed to be mainly responsible for interactions with RNAs, while CTR (~40 aa) forms an amyloid-like structure and reveals properties both related and unrelated to RNA metabolism [36,40,41,42,43]. Our previous analyses showed that Hfq, due to its amyloid-like C-terminal region, interacts in vitro with a lipid bilayer mimicking the *E. coli* inner membrane, producing holes in this membrane [19,20]. This observation raised the question of the presence of Hfq in the periplasmic space [19,20]. Indeed, the TEM localization of the Hfq protein suggested the presence of the protein in the periplasmic space (see Figure 2B,C of Diestra et al. [44]), but this could not be formally established due to the high resolution needed to localize a protein between the inner and outer membranes of a Gram-negative bacterium. Here, we show that Hfq is present in the OMV fraction, strongly suggesting that it is not only interacting with the inner membrane but that it is also exported in the periplasm. Indeed, while the mechanism of OMV biosynthesis remains unclear, it likely involves a breakage of the peptidoglycan, followed by an accumulation of macromolecules in the periplasm. Due to the outer membrane curvature (which could be induced by some proteins, possibly including Hfq, as suggested by our previous observations using solid-state NMR [20]), the OM is then blistered out and the OMV is formed [45]. As part of the molecules exported to the periplasm, sRNAs can be packed into OMVs and transported out of the cell, even if the mechanism by which sRNAs enter the vesicles is not fully understood. Here, we provide a new insight into this mechanism with Hfq being a part of the OMV’s cargo. This protein cannot only help sRNAs to cross the inner membrane, but can also protect them from degradation by RNAses [46], and finally it might help with the packaging of sRNAs into the OM.

**Figure 7:**
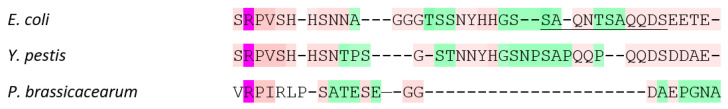
Sequence alignment of Hfq CTRs from *E. coli*, *Y. pestis*, and *P. brassicacearum*. In the alignment, strictly conserved R66 is indicated in hot pink; aa conserved in most Hfqs CTR are indicated in light pink. Other aa are indicated in green. As shown, the 11 amino acid residues of the amyloid region (underlined) are probably present in Hfq from *Y. pestis* but not in its homologue from *P. brassicacearum*.

Note that a similar analysis was performed with *Yersinia pestis* OMV [47] where the authors concluded that Hfq was not present in a significant amount in OMVs; nevertheless, traces of Hfq were present in the OMV fraction, especially in the strain devoid of the DplA protease [47]. Conversely, we could not find Hfq in *Pseudomonas brassicacearum* (unpublished result). This could be explained by the fact that *Y. pestis* Hfq shows sequence similarity to the amyloid-like region of *E. coli* Hfq, while *P. brassicacearum* Hfq does not share such a similarity (Figure 7). This is in agreement with our observation that Hfq needs its amyloid-like region to interact with and porate the membrane [19,20].

Finally, we also show that Hfq interacts with and inserts itself in the outer membrane, precisely in those OMV membranes that have the same composition as the OM. Again, the resolution needed to observe this in vivo is difficult to reach, but a previous TEM analysis also suggested this could be the case (see Figures 2B,C and 4B–D of Diestra et al. [44]). With this new analysis, we have now shown that Hfq may be present in the OM and may be exposed to the bacterial surface, as suggested by previous TEM images [44]. The finding that Hfq interacts and is inserted in the OMV membrane suggests that this protein may also be exposed at the cell surface and may help sRNA to be released into the OMV environment.

The presence of Hfq in OMVs, demonstrated in this report, has important biological implications. OMVs are used for communication between bacterial cells, and Hfq is an RNA chaperone which interacts with different regulatory sRNAs. These sRNA species have been shown to regulate mRNA translation via imperfect base-pairing, and Hfq is required to allow this regulatory process [48,49,50]. In fact, due to the diversity of their targets, sRNA and Hfq are involved in bacterial virulence and pathogenicity [51]. Therefore, we assume that Hfq included in OMV might be an element of the system facilitating various biological processes that require “cross-talks” between bacterial cells. Although the internalization of OMVs’ content, including RNAs, in the host cells has been observed [52], the precise mechanism is not understood yet (membrane fusion or endocytosis?); the subcellular localization(s) of internalized sRNAs in host cells is also undetermined. Knowing this would, however, have significant biological implications, especially in the regulation of various processes, including virulence and the resistance to antibiotics.

In this light, it is also worth mentioning that Hfq has been proposed as a potential target to fight different Gram-negative pathogenic bacteria [53]. Moreover, recent studies have demonstrated that it is involved in the *E. coli* response to antibiotics [54]. Interestingly, the effects of Hfq, and especially its CTR, were pronounced especially at high concentrations of antimicrobial compounds. Mutations in the *hfq* gene resulted in significant effects on the antibiotic resistance of *E. coli*, which was different depending on the localizations of the resistance genes, either in the chromosome or in plasmids. Moreover, it appeared that Hfq could modulate antibiotic resistance by both the regulation of the expression of antibiotic resistance genes, and the control of the replication of plasmids bearing such genes, thus modulating their copy numbers [55]. Under physiological conditions, this kind of regulation might be of special importance in the intestine, where local concentrations of orally administered antibiotics can be especially high, and in natural habitats if *E. coli* is present together with antibiotic-producing microorganisms. One might assume that cell-to-cell communication is very important in both the above-mentioned situations, and OMVs containing Hfq molecules might play important roles in facilitating the survival of *E. coli* cells. Apart from the regulation of the expression of genes coding for proteins responsible for antibiotic resistance, and the control of the copy number of plasmids bearing such genes, Hfq mediates drug resistance also by regulating the major efflux system AcrAB-TolC [56]. Therefore, it is plausible that Hfq present in OMV might not only regulate bacterial virulence but also modulate antibiotic resistance processes, supporting the importance of the phenomenon investigated and described in this study.

Finally, it is intriguing that OMVs of different bacteria were proposed as possible factors involved in the development of Alzheimer’s disease and other neurodegenerative disorders [57,58]. Moreover, it was suggested that bacterial amyloid-like proteins may stimulate human/animal β-amyloid accumulation by mechanisms involving the cross-seeding of misfolded proteins due to molecular mimicry [59]. Since Hfq contains the amyloid-like domain, one could speculate that its inclusion in OMVs produced by gut bacteria, like *E. coli*, might be an especially important risk factor for stimulating the development of neurodegenerative diseases in animals and humans.

## 4. Materials and Methods

### 4.1. Bacterial Strains

The bacterial strains used for OMV purification were MG1655 (the wild-type (WT) reference strain) and MG1655 *∆hfq*, which was described previously [36,54]. The *hfq* gene region of these strains was sequenced to confirm the presence of various *hfq* alleles (*hfq*^+^ or ∆*hfq*).

### 4.2. Production of Native and Truncated Forms of the Hfq Protein

Wild type *E. coli* Hfq was purified as described previously [60]. Its concentration was 8 mg/mL.

The Hfq-CTR peptide was chemically synthetized (Proteogenix, France) and prepared at 20 mg/mL, as described previously by Fortas et al. [18]. This peptide corresponds to the amyloid CTR domain of Hfq (residues 64 to 102) and its sequence is SRPVSHHSNNAGGGTSSNYHHGSSAQNTSAQQDSEETE.

### 4.3. Preparation of Outer Membrane Vesicles (OMV)

*OMVs extraction: E. coli* strains (WT or ∆*hfq*) were grown on Lysogeny Broth (LB) medium for 24 h (at 37 °C, 140 rpm). A volume of 300 mL of the culture was centrifuged twice at 8000× *g* for 30 min. The supernatant was then filtered through a 0.45 µm PES filter. This cell-free supernatant was ultracentrifuged at 150,000× *g* 4 °C for 2 h. The pellets obtained were re-suspended in 410 µL of sterile phosphate-buffered saline (PBS).

*OMVs purification:* OMVs were purified according to the density gradient purification method [61]. The gradient was composed of six phases prepared from the Optiprep 60% density gradient medium (Sigma Aldrich, Saint Louis, MO, USA): 45%, 40%, 35%, 30%, 25%, and 20%. The Optiprep medium was diluted with sterile PBS, except for the 45% fraction which was diluted directly with the OMVs sample. The gradient was centrifuged at 150,000× *g* at 4 °C for 20 h. The lipophilic fluorescent dye FM1-43 (Thermo Scientific, Waltham, MA USA) was used to quantify OMVs in each fraction and determine the concentration of OMVs. Then, a last centrifugation step was needed to separate OMVs from the Optiprep medium. Fractions concentrated in OMVs were centrifuged at 200,000× *g* for 2 h at 4 °C. The pellet was re-suspended in sterile PBS. The presence of OMVs was confirmed through light scattering experiments with a Zetasizer instrument (Malvern) and with the lipophilic fluorescent dye FM1-43 (5 µg/mL). The concentration of OMV vesicles is, however, difficult to estimate from this measurement.

### 4.4. Western Blotting Analysis

The OMV contents purified from WT *E. coli cells* were analyzed via Western blotting (WB). The WB membrane was revealed as described previously [62]; briefly, the membrane was successively incubated with a goat anti-Hfq polyclonal antibody for one hour at room temperature (dilution 1/1000, Origene, Herford, Germany). Then, after extensive washing, the goat antibody was revealed with an anti-goat secondary antibody coupled to alkaline phosphatase for one hour at room temperature (dilution 1/1000, Sigma), and was revealed with an NBT/BCIP solution (nitro blue tetrazolium chloride and 5-Bromo-4-Chloro-3-Indolyl-Phosphate, Sigma). We previously showed that the commercial antibodies for goat are specific using a Δ*hfq* strain [62].

### 4.5. Preparation of OM Supported Membranes for OCD Analysis

For the formation of a supported membrane, CaCl_2_ at a final concentration of 3 mM was added to the OMV solution. Then, 20 µL of the mix was put on a CaF_2_ surface cell and incubated for 1 h at RT. Then, the excess membranes were rinsed with dd-water five times to remove the excess OMV. The membrane was always kept wet in a chamber containing a saturated K_2_SO_4_ solution, giving a humidity of ~97%.

### 4.6. FTIR Analysis

Attenuated total reflection Fourier-transform infrared spectroscopy (ATR-FTIR) measurements were obtained with a diamond as an internal reflection element (IRE). A mixture of OMV extracted from the ∆*hfq* strain and Hfq or Hfq-CTR solutions was prepared. From this mixture, we took 2 µL of the sample, deposited it on the IRE, and dried it with a nitrogen flux at each kinetic timepoint. The system used was a Bruker Equinox55 purged with dry air, and a diamond ATR device with a single reflection at an angle of 45° and closed with a golden gate chamber from Specac (Orpington, UK). A spectrum, with a resolution of 4 cm^−1^, was acquired at different kinetics values. The data were treated with kinetics, a custom-made program developed in the SFMB laboratory (SFMB, Université libre de Bruxelles, Brussels, Belgique) running under MATLAB (Mathworks, Natick, MA, USA). The water vapor was removed by subtracting a reference spectrum of pure water vapor with a coefficient optimized on the amide II area band (1555–1550 cm^−1^). The spectra were normalized on the maximum of the amide I band.

### 4.7. Synchrotron Radiation Circular Dichroism (SRCD) and Orientated Circular Dichroism (OCD)

For SRCD analyses, the measurements and data collection were carried out on a DISCO beamline at Synchrotron SOLEIL (proposal 20210822) [63]. After different incubation times, 2–4 µL of samples were loaded onto circular demountable CaF_2_ cells of a 33-micron pathlength [64]. Three separated data collections with fresh sample preparations were carried out to ensure consistency and repeatability. Spectral acquisitions of 1 nm steps at a 1.2 s integration time, between 320 and 180 nm, were performed at 20 °C in triplicate for the samples as well as for the baselines. (+)-camphor-10-sulfonic acid (CSA) was used to calibrate the amplitudes and wavelength positions of the SRCD experiment. Data analyses including averaging, baseline subtraction, smoothing, scaling, and standardization were carried out using CDtoolX [65]. Secondary structure contents were determined using BeStSel [33].

For OCD, OMVs were deposited onto the CaF_2_ cell surface. Hfq or CTR solutions were then dispensed on top. Lipid films must be thin to minimize scattering and assure homogeneity. A CaF_2_ lid was put on top (20 µm pathlength), preserving the humidity within the closed cell, and keeping the membrane/protein sample airtight. The OCD spectra were averaged by recording the CD spectra of the beam-centered cell, with the membrane/protein layer circulating clockwise every 45° using an automated rotating chamber [66]. This method allowed for the elimination of linear dichroism due to the alignment of the peptides in or on the membrane, resulting in an OCD spectrum, as described by Chen [67].

## 5. Conclusions

Evidence is provided showing that the Hfq riboregulator interacts not only with the inner membrane, but also with the outer membrane of *E. coli*. This opens a new way to understand its unexpected function in facilitating sRNAs to be packaged in OMVs [6]. Indeed, the precise molecular mechanism of the formation of sRNA-containing OMVs is still unproven [68]. Nevertheless, with this newly identified property of Hfq, one can speculate that this protein interacts with the inner membrane, and that it can be translocated into the periplasm; then, in a second step, OMVs might form and trap Hfq either inside the vesicle or in the outer membrane that forms OMVs via budding. Finally, as a well-known sRNA stabilizer [46], Hfq may help to stabilize sRNA when exported outside the cell in the OMV. This would have significant biological implications, especially in the communication between bacterial cells and the regulation of various processes, including those related to bacterial virulence and the resistance to antibiotics.

## Data Availability

The data that support the findings of this study are available on request from the corresponding authors.

## Supplementary Materials

The following supporting information can be downloaded at https://www.mdpi.com/article/10.3390/ijms241411424/s1.
