# Supplementary figures and images for: Interactions and Insertion of Escherichia coli Hfq into Outer Membrane Vesicles as Revealed by Infrared and Orientated Circular Dichroism Spectroscopies
Source: Int J Mol Sci. 2023 Jul 13;24(14):11424. doi: 10.3390/ijms241411424 (PMC10379585; doi:10.3390/ijms241411424)

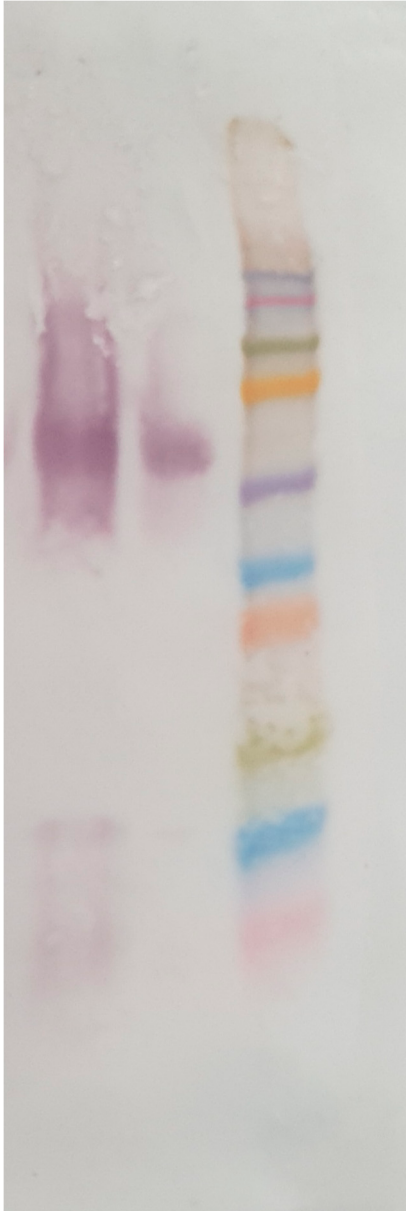

Figure S1: OMV extracted from  $\Delta hfq$  strain observed by TEM (negative 425 staining).

Supplement: Supplementary file 1 [file ijms-24-11424-s001.zip › ijms-2501268-supplementary.pdf]
